# Tooth loss and infrequent brushing significantly elevate gastric cancer risk: an updated systematic review and meta-analysis with heterogeneity exploration

**DOI:** 10.3389/fmed.2026.1761829

**Published:** 2026-02-27

**Authors:** Tangna Ge, Pingping Cui, Yuxin Ma, Xiaoyan Li, Shuo Wu

**Affiliations:** 1 Department of Endodontics, School and Hospital of Stomatology, Cheeloo College of Medicine, Shandong University & Shandong Key Laboratory of Oral Tissue Regeneration & Shandong Engineering Research Center of Dental Materials and Oral Tissue Regeneration & Shandong Provincial Clinical Research Center for Oral Diseases, Jinan, China; 2 Department of Stomatology, The First People's Hospital of Jinan, Jinan, China; 3 Department of Emergency Medicine, Qilu Hospital of Shandong University, Jinan, China; 4 Shandong Provincial Clinical Research Center for Emergency and Critical Care Medicine, NMPA Key Laboratory for Clinical Research and Evaluation of Innovative Drug, Medical and Pharmaceutical Basic Research Innovation Center of Emergency and Critical Care Medicine, China's Ministry of Education, Shandong Provincial Engineering Laboratory for Emergency and Critical Care Medicine, Key Laboratory of Cardiopulmonary-Cerebral Resuscitation Research of Shandong, Qilu Hospital of Shandong University, Jinan, China

**Keywords:** gastric cancer, meta-analysis, systematic review, tooth brushing, tooth loss

## Abstract

**Background:**

The association between oral hygiene—specifically, tooth brushing and tooth loss—and gastric cancer (GC) incidence risk remains inconclusive. Previous meta-analyses are outdated and insufficient in exploring heterogeneity. To address this gap, this study aims to perform an updated and comprehensive evaluation of this association.

**Methods:**

We systematically searched PubMed, Embase, Web of Science, Scopus, and the Cochrane Library up to April 2024 for studies examining tooth brushing frequency, tooth loss, and GC risk. Pooled odds ratios (ORs) with 95% confidence intervals (CIs) were calculated using a random-effects model. Heterogeneity was assessed and explored via meta-regression and subgroup analysis.

**Results:**

Twelve studies were included. Infrequent tooth brushing was associated with a 55% increased GC risk (OR 1.55, 95% CI: 1.17–2.05). Despite significant heterogeneity, largely attributable to study design, this association was robust in sensitivity analyses. Tooth loss also significantly increased GC risk (OR 1.25, 95% CI: 1.12–1.39). Notably, subgroup analyses identified geographic region as a key source of heterogeneity, with the tooth loss-associated risk being substantially stronger in Europe and America (OR 1.68) than in Asia (OR 1.19) populations. Sensitivity analyses supported the robustness of these findings.

**Conclusion:**

This study solidifies poor oral hygiene as a significant risk factor for GC. The marked geographic disparity offers novel insights for developing targeted primary prevention strategies, underscoring the importance of integrating oral health into general cancer prevention initiatives.

**Systematic review registration:**

http://www.crd.york.ac.uk/prospero, identifier: CRD42024546658.

## Introduction

Gastric cancer (GC) remains a formidable global health challenge, being the third leading cause of cancer-related disability-adjusted life years worldwide (1). In several regions, it is the foremost cause of cancer incidence and mortality, underscoring the urgent need to identify modifiable risk factors for primary prevention (1).

Beyond established risk factors such as *H. pylori* infection and dietary habits, the role of oral hygiene has garnered increasing interest. Poor oral hygiene, characterized by infrequent tooth brushing and subsequent tooth loss, may serve as a reservoir for oral pathogens and promote the endogenous formation of carcinogens, such as nitrosamines, thereby potentially influencing gastric carcinogenesis (2–5). While numerous observational studies have investigated the links between tooth brushing frequency, tooth loss, and GC risk, the findings have been inconsistent and contentious (6–9). Some reports suggest a significant positive association, whereas others have yielded non-significant or discordant results, leaving the field in a state of uncertainty.

Two previous meta-analyses have attempted to synthesize this evidence but with critical limitations. An analysis conducted 8 years ago identified tooth loss as a potential risk marker for GC, but considerable heterogeneity across the studies precluded a definitive conclusion (6). Another, more recent analysis found no statistically significant association for tooth brushing frequency or tooth loss with GC, likely due to its broader focus on oral health and the limited number of studies available for these specific exposures at the time (7). Consequently, a definitive conclusion regarding the independent and combined roles of these simple, measurable oral hygiene indicators in GC development has not been reached.

Given the accumulation of new epidemiological data in recent years and the persistent controversy in the field, an updated, comprehensive, and rigorously conducted synthesis is imperative. The present systematic review and meta-analysis aims to: (1) provide the most contemporary and precise estimates of the associations between tooth brushing frequency, tooth loss, and GC incidence; and (2) explore potential sources of heterogeneity through extensive subgroup and meta-regression analyses (e.g., by geographic region, study design). Our findings are expected to clarify this clinically relevant association and inform future public health initiatives aimed at GC prevention through the promotion of good oral hygiene.

## Materials and methods

### Literature search

The systematic literature search was performed in PubMed, Embase, Web of Science, Cochrane Library and Scopus for relevant articles published in English from inception until April 2024. The search strategy for oral health (tooth brushing frequency and tooth loss included: “tooth loss” OR “teeth loss” OR “toothbrushing” OR “tooth brushing” OR “teeth brushing” OR “missing teeth” OR “tooth missing” OR “tooth behavior” OR “oral hygiene” OR “oral health”. For GC, the terms were: “stomach cancer” OR “gastric cancer” OR “gastric non-cardia adenocarcinoma” OR “gastric cardia adenocarcinoma” OR “gastric adenocarcinoma” OR “stomach neoplasms”. The two sets of terms were combined using the Boolean operator “AND”. The search was restricted to studies in English involving human subjects. Complete search strategies are detailed in Supplementary Table S1—Supplementary Table S5. Additionally, the reference lists of all eligible articles were checked to identify further pertinent studies. This meta-analysis was conducted in accordance with the Preferred Reporting Items for Systematic reviews and Meta-Analysis (PRISMA) guidelines (10) (Supplementary Table S6) and was prospectively registered on PROSPERO (http://www.crd.york.ac.uk/prospero, CRD42024546658).

### Eligibility criteria

Two investigators independently screened the titles and abstracts of retrieved records against the pre-defined eligibility criteria. Discrepancies were resolved through discussion or by consulting a third reviewer. The full texts of potentially relevant articles were then independently assessed by the same two reviewers.

The inclusion criteria were as follows:

a. Studies performed in humans.

b. Case-control or cohort study design.

c. The outcome was the GC incidence.

d. The sample size, odds ratios (OR) and 95% confidence intervals (CIs), or hazard ratios (HR) and 95% CIs was reported.

The exclusion criteria were as follows:

a. Studies reporting on duplicate datasets.

b. Meta-analysis, review, editorial or letter.

c. Articles not published in English.

### Data extraction and quality assessment

The following data were extracted from each included study: first author, publication year, number of events, sample size, geographic region (Asia, Europe and America), the study design (case-control, cohort), population characteristics, anatomic subtype (cardia, non-cardia), method of outcome ascertainment, effect measures (OR/HR and 95% CIs), and the adjusted confounders, etc. If multiple reports from the same population were available, only the estimate from the largest sample size or longest follow-up period was extracted.

For both tooth loss and tooth brushing frequency, exposures were categorized into two groups for analysis: the group with the highest exposure (e.g., most tooth lost or least frequent brushing) was compared against the reference group with the lowest exposure (e.g., least tooth lost or most frequent brushing).

The Newcastle–Ottawa scale (NOS) was used to evaluate the quality of the included studies, assessing three domains: selection, comparability, and exposure/outcomes (11, 12). The total scale ranges from 0 to 9 points. Studies were categorized as high (≥7 points), moderate (4–6 points) and low quality (0–3 points). Data extraction and quality assessments were performed independently by two reviewers, with disagreements resolved by the third reviewer.

### Statistical analysis

Summary effect estimates for the contributions of tooth brushing and tooth loss to GC incidence were calculated as ORs and 95% CIs with the inverse variance -weighted mean of the logarithm of the risk ratios. Between-study heterogeneity was quantified using Higgins *I*^2^ statistic (13), where an *I*^2^ value of >50% was considered to represent substantial heterogeneity. Given the anticipated clinical and methodological diversity across studies, the random effects model (REM) recommended by DerSimonian and Laird was applied to all meta-analytic outcomes, regardless of heterogeneity (14).

Publication bias was assessed visually though funnel plot and formally tested with Egger linear regression tests and Begg's test (15). The ‘leave one out' sensitivity analysis was performed to evaluate the influence of individual studies on between-study heterogeneity (16).

To explore potential sources of heterogeneity and the effects of study characteristics on the overall estimates, we conducted pre-specified subgroup analyses and meta-regressions based on the following study-level characteristics: study design (case-control vs. cohort), anatomic subtype (cardia vs. non-cardia), geographic region (Asia vs. Europe and America), adjustment for smoking (yes vs. no), and study quality (NOS score >6 vs. ≤ 6).

All analyses were performed using R statistical software (version 4.2.2, R Foundation for Statistical Computing, Vienna, Austria) with meta-packages. A two-tailed *P* value of <0.05 was considered statistically significant.

## Results

### Study selection and characteristic

**Figure 1:**
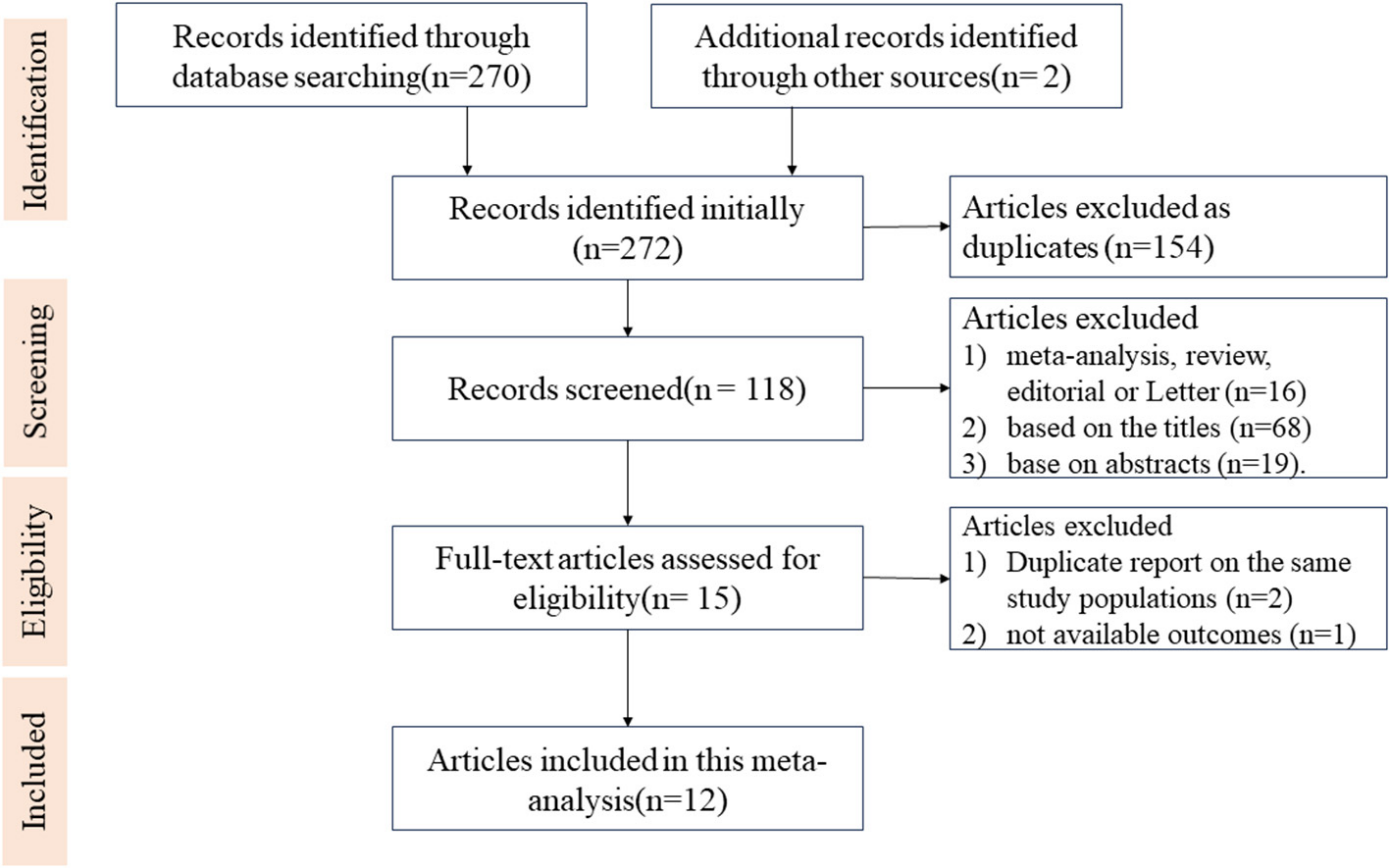
Flow diagram of study based on the eligibility criteria.

Our systematic search identified 272 unique records. Following the PRISMA guidelines (Figure 1), 154 duplicates and 16 publications that were meta-analyses, reviews, editorials or letters were excluded. Screening of titles and abstracts led to the exclusion of 68 and 19 records, respectively. Fifteen articles underwent full-text review. One study was excluded due to unavailable outcomes data (17). For overlapping populations reported in multiple publications (18–21), we included the study with the larger sample size or longer follow-up period for each unique cohort in the final quantitative synthesis (19, 21). Additionally, two studies reported on tooth loss in Korean National Health Screening Cohort (22, 23), the result from the larger population although with shorter follow-up period was included in the final analysis (23).

Ultimately, twelve studies were included in the meta-analysis (Supplementary Table S7). The characteristics comprised four case-control and eight cohort studies, with nine conducted in Asia and three in America or Europe. Most studies provided risk estimates adjusted for key confounders including age (all 12 studies), sex (8 studies), and smoking (8 studies). Quality assessment using the Newcastle Ottawa Scale, revealed that all cohort studies and one case-control study were of high quality (score ≥7), while the remaining three case-control studies were of moderate quality (Supplementary Table S8,S9).

### Association between Tooth Brushing and Gastric Cancer

**Figure 2:**
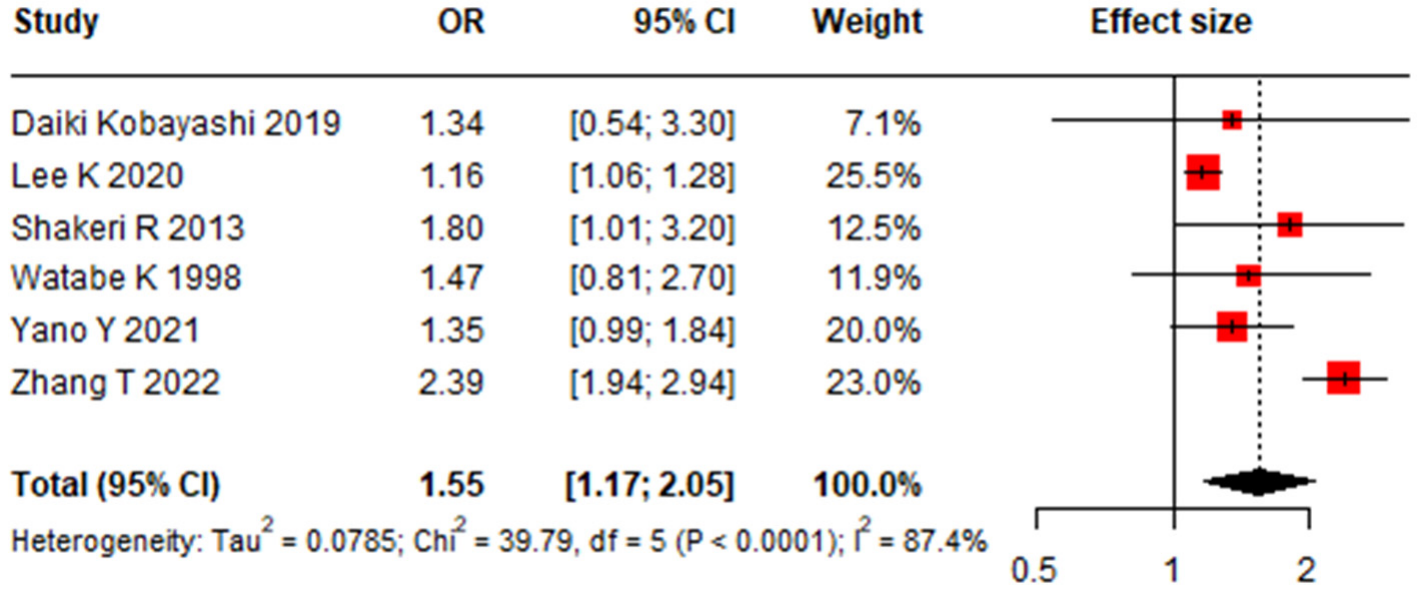
Forest plot for the association between tooth brushing and gastric cancer risk.

**Figure 3:**
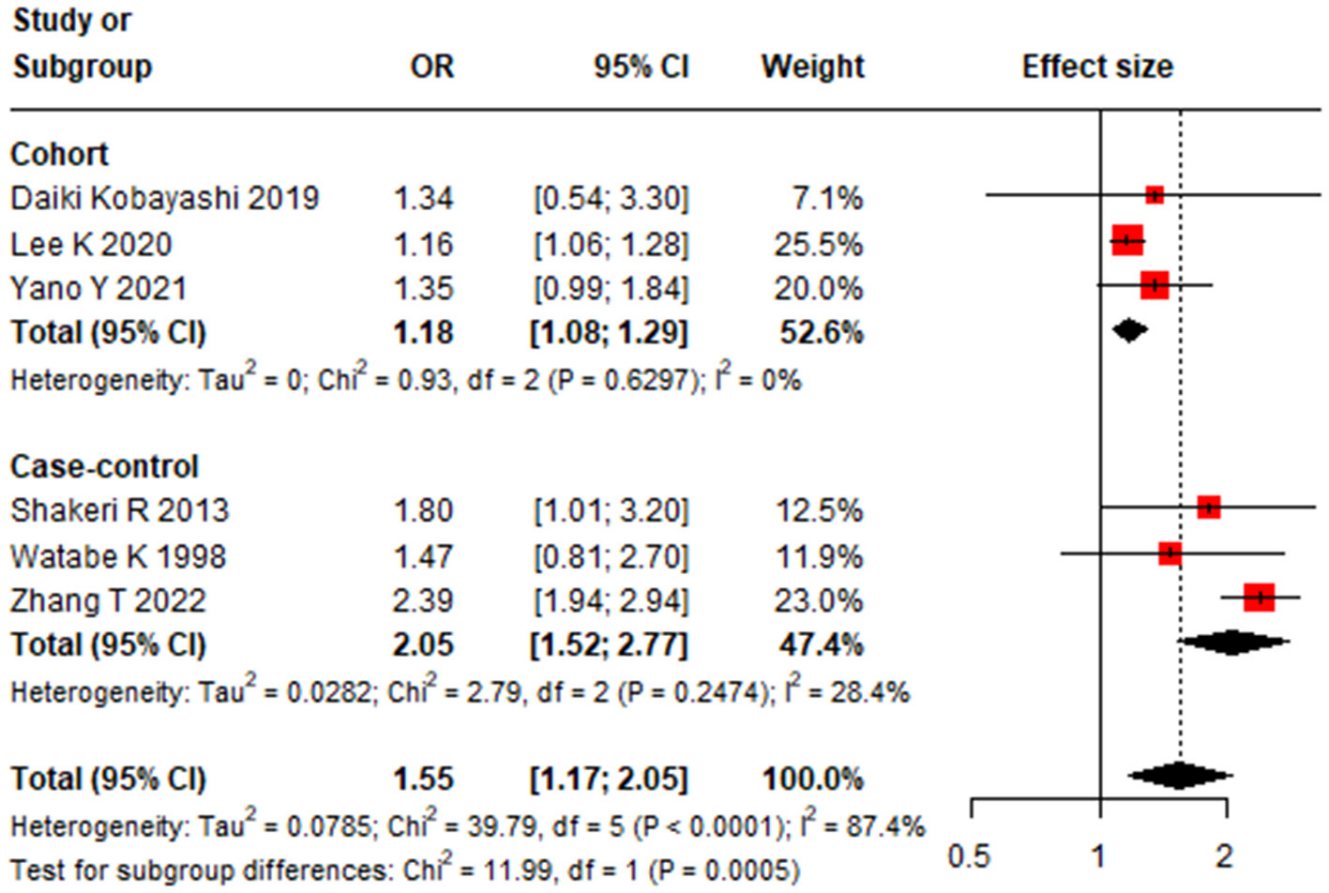
Subgroup-analysis results of cohort, and case-control study for the association between tooth brushing and gastric cancer.

Infrequent tooth brushing was significantly associated with an increased risk of GC, with a pooled OR of 1.55 (95% CI: 1.17–2.05) (22, 24–28) (Figure 2). Significant heterogeneity was observed among these studies (*I*^2^ = 87.4%, 95% CI: 75.0%−93.7%, *P* < 0.01). Meta-regression identified study design as a significant source of this heterogeneity (*P* < 0.01) (Table 1). In subgroup analysis, the association remained significant in both case-control studies (OR 2.05, 95% CI 1.52–2.77) and cohort studies (OR 1.18, 95% CI 1.08–1.29) (Table 1, Figure 3). No single study unduly influenced the overall result in sensitivity analysis (Supplementary Figure S2). Both Egger linear regression test (*P* = 0.395) and Begg's test (*P* = 0.999) suggested no evidence of publication bias (Supplementary Figure S1).

**Table 1:** Results of overall and subgroup analyses of pooled ORs and 95% CIs.

**Studies**	**No. of studies**	Pooled estimate effect		Test of homogeneity		meta regression	
		**OR**	**(95% CI)**	*I*^2^ **(%)**	***P*** **Value**	**I**^2^ **(%)**	***P*** **Value**
**Tooth brushing**			
**Design**			
Cohort	3	1.18	1.08–1.29	0	0.63	95.28	<0.01
Case–control	3	2.05	1.52–2.77	28	0.25		
**Adjusted smoking**			
No	5	1.73	1.29–2.32	62	0.03	26.72	0.17
Yes	1	1.16	1.06–1.28	—	—		
**NOS**			
>=7	4	1.52	1.04–2.22	92	<0.01	0	0.85
<7	2	1.63	1.08–2.48	0	0.63		
**Tooth loss**			
**Design**			
Cohort	6	1.25	1.12–1.40	49	0.03	0	0.72
Case–control	4	1.21	0.88–1.66	59	0.06		
**The anatomic subtypes**			
Gastric	8	1.34	1.12–1.61	59	0.01	0	0.70
Non–cardia	2	1.22	1.01–1.48	0	0.43		
cardia	1	1.18	0.92–1.50	69	0.004		
**Adjusted smoking**			
No	5	1.43	1.12–1.82	47	0.11	7.6	0.19
Yes	5	1.19	1.07–1.31	47	0.04		
**NOS**			
> = 7	7	1.23	1.11–1.36	47	0.03	0	0.66
<7	3	1.30	0.83–2.04	62	0.07		
**Geographic region**			
Europe and America	3	1.68	1.22–2.23	53	0.09	35.1	0.03
Asian	7	1.19	1.07–1.31	33	0.13		

### Association between tooth loss and gastric cancer

**Figure 4:**
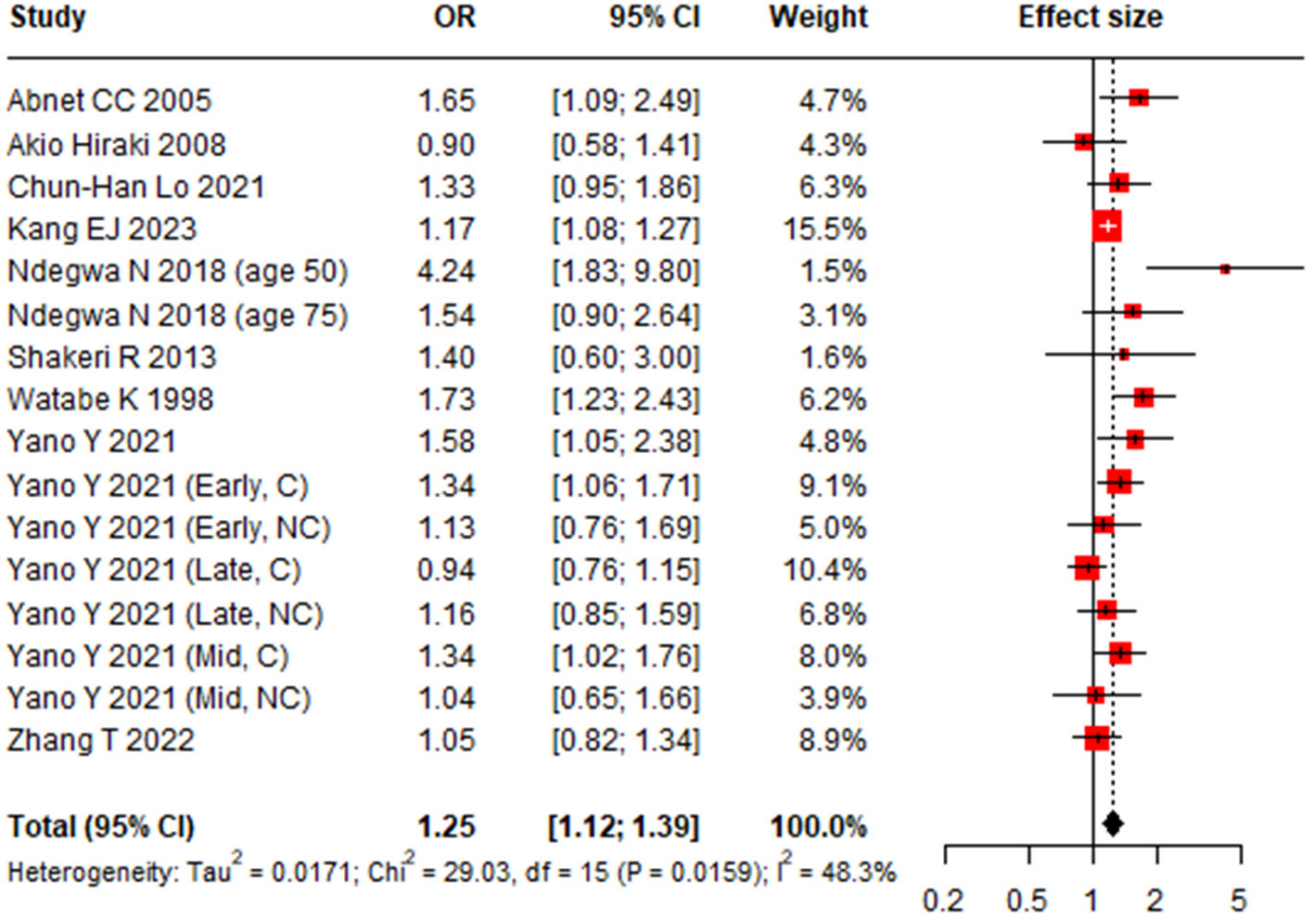
Forest plot for the association between tooth loss and gastric cancer risk.

**Figure 5:**
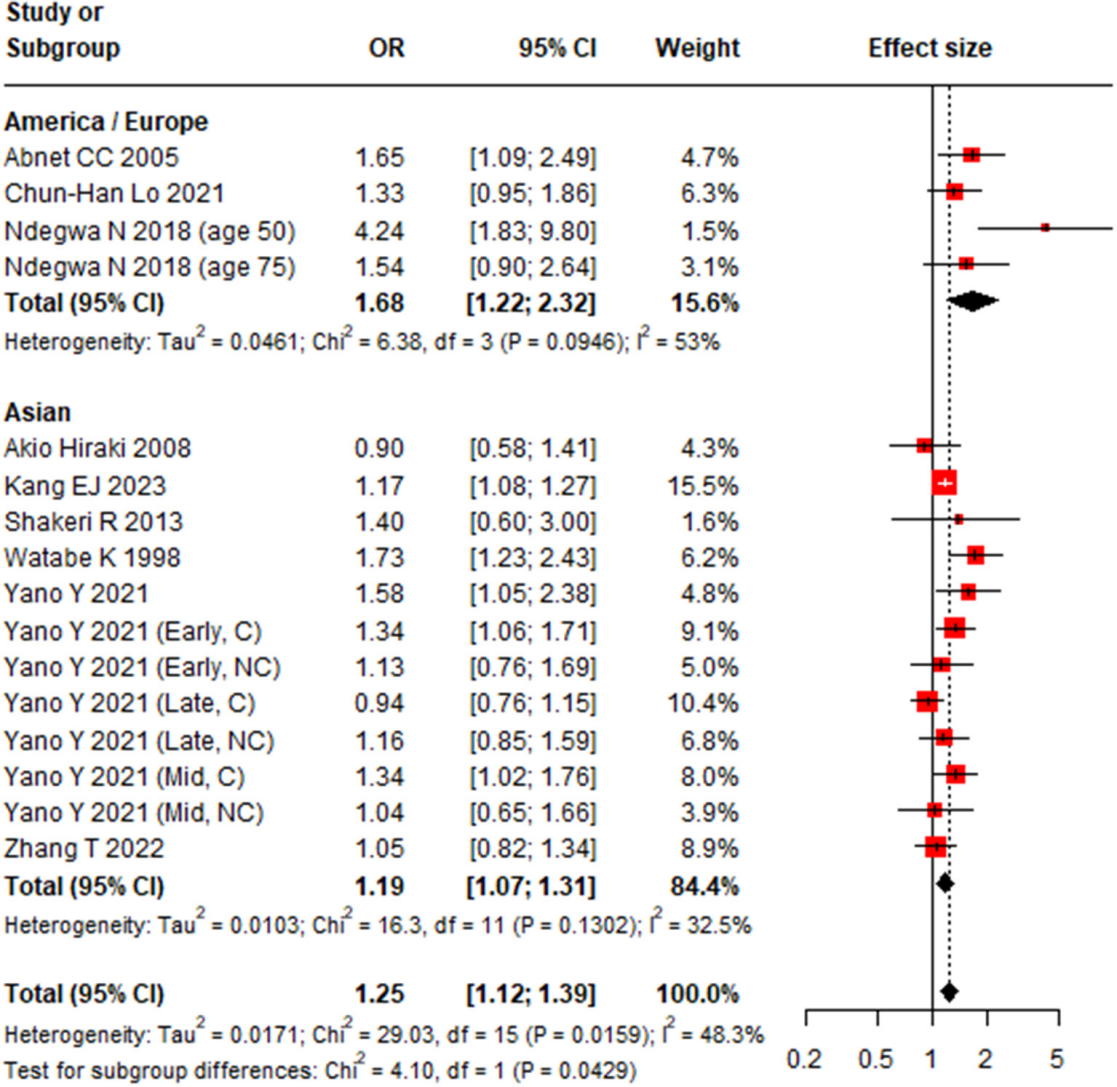
Subgroup-analysis results of geographic region for the association between tooth brushing and gastric cancer risk.

Pooled analysis demonstrated a significant association between tooth loss and GC occurrence (OR 1.25, 95% CI, 1.12–1.39) (19, 21, 23–27, 29–31) (Figure 4) with moderate heterogeneity (*I*^2^ = 48.3%, 95%CI: 7.8%−71.0%, *P* < 0.02). Meta-regression identified geographic region (*P* = 0.03) as a potential source of heterogeneity (Table 1). Subgroup analyses revealed a stronger association in studies from Europe and America (OR 1.68, 95% CI: 1.22–2.32) than in those from Asia (OR 1.19, 95% CI: 1.07–1.31) (Table 1, Figure 5). Notably, the association was not statistically significant in the subgroup of case-control studies, cardia cander, or studies with NOS score less than 7. Sensitivity analysis confirmed the robustness of the pooled estimate (Supplementary Figure S4), and no publication bias was detected (Egger linear regression test *P* = 0.105; Begg's test *P* = 0.163) (Supplementary Figure S3).

## Discussion

This comprehensive meta-analysis provides robust evidence that poor oral hygiene, characterized by infrequent tooth brushing and tooth loss, is significantly associated with an increased risk of gastric cancer (GC). The magnitude of the association was particularly strong for infrequent brushing, with a 55% increased risk, while tooth loss was associated with a 25% increased risk. These findings underscore the importance of oral health as a modifiable risk factor in gastric carcinogenesis.

The association between infrequent tooth brushing and GC, though supported by our pooled analysis, has been less explored and marked by inconsistencies in prior literature. All six (22, 24–28) identified studies were conducted in Asia, and the association was primarily driven by case-control studies, which are more susceptible to recall bias. However, the robustness of our pooled estimate, reinforced by consistent findings across key subgroups (e.g., adjusted for smoking, high-quality studies), strengthens the argument for a causal link. The stronger effect size in case-control studies may indeed reflect this methodological bias, but the persistent, albeit weaker, association in prospective cohort designs suggests an underlying biological effect.

Our study confirms the positive association between tooth loss and GC previously reported, but with a critical refinement. We identified geographic region as a key effect modifier, with a significantly stronger association observed in Europe and America (OR = 1.68) compared to Asian (OR = 1.19). This intriguing finding may be explained by the differing etiological landscape of GC (6). In high-incidence regions like East Asia, where dominant risk factors such as *H. pylori* infection and high salt intake are pervasive, the relative contribution of oral hygiene might be attenuated. In contrast, in lower-incidence Europe and America populations, oral health may emerge as a more discernible and independent risk factor. This hypothesis-generating finding warrants further investigation

The association is supported by several plausible biological pathways that form an “oral-gastric axis” of carcinogenesis:

Microbial Carcinogenesis: Poor oral hygiene fosters a dysbiotic microbiome, enriching for periodontal pathogens and facilitating the colonization and persistence of Helicobacter pylori in the oral cavity (32–35). This serves as a reservoir for gastric reinfection or increased microbial load, driving chronic gastritis and carcinogenesis (4, 5, 36).

Chronic Inflammation: Periodontitis is a state of persistent, low-grade systemic inflammation (5). Inflammatory mediators (e.g., IL-1β, TNF-α, PGE2) released from periodontal tissues can reach the gastric mucosa via circulation, potentially synergizing with local inflammatory processes to promote a pro-carcinogenic microenvironment.

Nitrosamine Production: The oral bacterial flora is instrumental in converting of dietary nitrates to nitrites, which can then form N-nitroso compounds (NOCs) in the stomach (37, 38). Many NOCs are potent genotoxic carcinogens, and their increased production due to oral dysbiosis represents a direct chemical link to gastric cancer (39, 40).

Nutritional and Functional Compromise: Extensive tooth loss impairs masticatory function, leading to a dietary shift toward softer, often less nutritious, and potentially more carcinogenic foods. This can result in deficiencies of protective antioxidants and vitamins, while the consumption of poorly chewed, coarse food might cause mechanical irritation to the gastric mucosa (41).

From a clinical standpoint, our findings suggest that a patient's oral health history can serve as a simple, low-cost risk indicator for GC. Gastroenterologists and primary care physicians may consider incorporating questions about tooth brushing habits and tooth loss into routine risk assessments, particularly for high-risk individuals (2).

From a public health perspective, these results are highly significant. Promoting good oral hygiene represents a low-risk, cost-effective strategy that could be integrated into national GC prevention programs. Public health messaging should emphasize that brushing tooth is not only about preventing cavities and gum disease but may also be a vital component in reducing the risk of serious systemic conditions, including gastric cancer. This aligns with the WHO's emphasis on integrating oral health into general health promotion (42).

The primary strength of this study lies in its comprehensive and rigorous methodology, representing the most up-to-date and thorough synthesis on this topic to date. However, several limitations must be acknowledged. First, the restriction to English-language publications may have introduced selection bias. Second, the high heterogeneity, particularly for tooth brushing, although explored, indicates the influence of unmeasured confounding factors. Third, exposure assessment varied across studies, and residual confounding by factors like socioeconomic status, detailed oral hygiene practices (e.g., use of, mouthwash, dental floss, brushing time, electric toothbrush), and dietary habits remain possible. Fourth, the combined effects of lifestyle factors, particularly smoking, and poor oral hygiene could not be assessed due to a lack of stratified data. Fifth, our subgroup analysis by geographic region (Asia vs. Europe and America) could not account for ethnic diversity. As the original studies did not report ethnicity-stratified estimates, we were unable to assess whether the observed association differs among groups such as African American, Hispanic, or Asian American populations.

## Conclusion

In conclusion, this meta-analysis solidifies the link between poor oral hygiene—manifested as infrequent tooth brushing and tooth loss—and an increased risk of gastric cancer. These findings highlight the importance of oral hygiene not merely as a goal of dental care, but as an integral component of holistic gastrointestinal health and primary cancer prevention. Future prospective studies with standardized oral health assessments are encouraged to validate these findings and elucidate the precise mechanisms at play.

## Data Availability

The original contributions presented in the study are included in the article/Supplementary material, further inquiries can be directed to the corresponding authors.
