# Supplementary material for: Tooth loss and infrequent brushing significantly elevate gastric cancer risk: an updated systematic review and meta-analysis with heterogeneity exploration
Source: Front Med (Lausanne). 2026 Feb 27;13:1761829. doi: 10.3389/fmed.2026.1761829 (PMC12983529; doi:10.3389/fmed.2026.1761829)
Supplement: Supplementary file 1 [file Data_Sheet_1.docx]

**S1 Table Search strategies in Pubmed**

|  | **Search terms** | **No. of articles** |
| --- | --- | --- |
| **#1** | “stomach cancer” [All Fields] OR “gastric cancer” [All Fields] OR “gastric non-cardia adenocarcinoma” [All Fields] OR “gastric cardia adenocarcinoma” [All Fields] OR “Gastric adenocarcinoma” [All Fields] OR “Stomach Neoplasms” [All Fields] | [142,585](https://pubmed.ncbi.nlm.nih.gov/?term=%E2%80%9Cstomach+cancer%E2%80%9D+%5BAll+Fields%5D+OR+%E2%80%9Cgastric+cancer%E2%80%9D+%5BAll+Fields%5D+OR+%E2%80%9Cgastric+non-cardia+adenocarcinoma%E2%80%9D+%5BAll+Fields%5D+OR+%E2%80%9Cgastric+cardia+adenocarcinoma%E2%80%9D+%5BAll+Fields%5D+OR+%E2%80%9CGastric+adenocarcinoma%E2%80%9D+%5BAll+Fields%5D+OR+%E2%80%9CStomach+Neoplasms%E2%80%9D+%5BAll+Fields%5D&size=200&sort=relevance) |
| **#2** | “tooth loss” [All Fields] OR “teeth loss” [All Fields] OR “toothbrushing” [All Fields] OR “tooth brushing” [All Fields] OR “teeth brushing” [All Fields] OR “missing teeth” [All Fields] OR “tooth missing” [All Fields] OR “tooth behavior” [All Fields] OR “oral hygiene” [All Fields] OR “oral health” [All Fields] | 98,282 |
| **Final strategy** | **#1** AND **#2** Filters: **Humans, English** | 49 |

**S2 Table** **Search strategies in Embase**

|  | **Search terms** | **No. of articles** |
| --- | --- | --- |
| **#1** | “stomach cancer” OR “gastric cancer” OR “gastric non-cardia adenocarcinoma” OR “gastric cardia adenocarcinoma” OR “Gastric adenocarcinoma” OR “Stomach Neoplasms” | 169,943 |
| **#2** | “tooth loss” OR “teeth loss” OR “toothbrushing” OR “tooth brushing” OR “teeth brushing” OR “missing teeth” OR “tooth missing” OR “tooth behavior” OR “oral hygiene” OR “oral health” | 99,046 |
| **Final strategy** | **#1 AND #2 AND [english]/lim AND [humans]/lim** | 91 |

**S3 Table Search strategies in Web of Science**

|  | **Search terms** | **No. of articles** |
| --- | --- | --- |
| **#1** | ALL=(“stomach cancer” OR “gastric cancer” OR “gastric non-cardia adenocarcinoma” OR “gastric cardia adenocarcinoma” OR “Gastric adenocarcinoma” OR “Stomach Neoplasms”) | 116,513 |
| **#2** | “tooth loss” OR “teeth loss” OR “toothbrushing” OR “tooth brushing” OR “teeth brushing” OR “missing teeth” OR “tooth missing” OR “tooth behavior” OR “oral hygiene” OR “oral health” | 59,130 |
| **Final strategy** | **#1** AND **#2** NOT “Review” [Publication Type] NOT “Editorial” [Publication Type] NOT “Letter” [Publication Type] AND “Humans” [Filter] | 67 |

### S4 Table Search strategies in Scopus

|  | **Search terms** | **No. of articles** |
| --- | --- | --- |
| **#1** | TITLE-ABS-KEY ( "stomach cancer" OR "gastric cancer" OR "gastric non-cardia adenocarcinoma" OR "gastric cardia adenocarcinoma" OR "Gastric adenocarcinoma" OR "Stomach Neoplasms" ) | 159,513 |
| **#2** | TITLE-ABS-KEY ( "tooth loss" OR "teeth loss" OR "toothbrushing" OR "tooth brushing" OR "teeth brushing" OR "missing teeth" OR "tooth missing" OR "tooth behavior" OR "oral hygiene" OR "oral health" ) | 91,784 |
| **Final strategy** | **#1** AND **#2** AND “Article” [Filter] AND "English" [Filter] | 56 |

### S5 Table Search strategies in Cochrane Library

|  | **Search terms** | **No. of articles** |
| --- | --- | --- |
| **#1** | "stomach cancer" OR "gastric cancer" OR "gastric non-cardia adenocarcinoma" OR "gastric cardia adenocarcinoma" OR "Gastric adenocarcinoma" OR "Stomach Neoplasms" | 22 |
| **#2** | "tooth loss" OR "teeth loss" OR "toothbrushing" OR "tooth brushing" OR "teeth brushing" OR "missing teeth" OR "tooth missing" OR "tooth behavior" OR "oral hygiene" OR "oral health" | 215 |
| **Final strategy** | **#1** AND **#2** NOT “Review” [Publication Type] NOT “Editorial” [Publication Type] NOT “Letter” [Publication Type] NOT Humans [Filter] | 7 |

**S6 Table. Prisma checklist.**

| **Section and Topic** | **Item #** | **Checklist item** | **Location where item is reported** |
| --- | --- | --- | --- |
| **TITLE** | | |  |
| Title | 1 | Identify the report as a systematic review. | P1: Title |
| **ABSTRACT** | | |  |
| Abstract | 2 | See the PRISMA 2020 for Abstracts checklist. | P2: Abstract |
| **INTRODUCTION** | | |  |
| Rationale | 3 | Describe the rationale for the review in the context of existing knowledge. | P4: Introduction |
| Objectives | 4 | Provide an explicit statement of the objective(s) or question(s) the review addresses. | P5: Introduction |
| **METHODS** | | |  |
| Eligibility criteria | 5 | Specify the inclusion and exclusion criteria for the review and how studies were grouped for the syntheses. | P6: Materials and Methods (Eligibility criteria) |
| Information sources | 6 | Specify all databases, registers, websites, organisations, reference lists and other sources searched or consulted to identify studies. Specify the date when each source was last searched or consulted. | P5: Materials and Methods (Literature search) |
| Search strategy | 7 | Present the full search strategies for all databases, registers and websites, including any filters and limits used. | P5 Materials and Methods (Literature search); S1 – S5 Tables |
| Selection process | 8 | Specify the methods used to decide whether a study met the inclusion criteria of the review, including how many reviewers screened each record and each report retrieved, whether they worked independently, and if applicable, details of automation tools used in the process. | P6: Materials and Methods (Eligibility criteria) |
| Data collection process | 9 | Specify the methods used to collect data from reports, including how many reviewers collected data from each report, whether they worked independently, any processes for obtaining or confirming data from study investigators, and if applicable, details of automation tools used in the process. | P6-P7: Materials and Methods (Data extraction and quality assessment) |
| Data items | 10a | List and define all outcomes for which data were sought. Specify whether all results that were compatible with each outcome domain in each study were sought (e.g. for all measures, time points, analyses), and if not, the methods used to decide which results to collect. | P7: Materials and Methods (Data extraction and quality assessment) |
|  | 10b | List and define all other variables for which data were sought (e.g. participant and intervention characteristics, funding sources). Describe any assumptions made about any missing or unclear information. | P6: Materials and Methods (Data extraction and quality assessment) |
| Study risk of bias assessment | 11 | Specify the methods used to assess risk of bias in the included studies, including details of the tool(s) used, how many reviewers assessed each study and whether they worked independently, and if applicable, details of automation tools used in the process. | P7: Materials and Methods (Data extraction and quality assessment) - NOS Scale |
| Effect measures | 12 | Specify for each outcome the effect measure(s) (e.g. risk ratio, mean difference) used in the synthesis or presentation of results. | P7: Materials and Methods (Statistical analysis) - ORs |
| Synthesis methods | 13a | Describe the processes used to decide which studies were eligible for each synthesis (e.g. tabulating the study intervention characteristics and comparing against the planned groups for each synthesis (item #5)). | P7: Materials and Methods (Data extraction and quality assessment) - Exposure categorization |
|  | 13b | Describe any methods required to prepare the data for presentation or synthesis, such as handling of missing summary statistics, or data conversions. | P6: Materials and Methods (Data extraction and quality assessment) - Handling of multiple reports |
|  | 13c | Describe any methods used to tabulate or visually display results of individual studies and syntheses. | P7: Materials and Methods (Statistical analysis) - Forest plots, etc. |
|  | 13d | Describe any methods used to synthesize results and provide a rationale for the choice(s). If meta-analysis was performed, describe the model(s), method(s) to identify the presence and extent of statistical heterogeneity, and software package(s) used. | P7: Materials and Methods (Statistical analysis) |
|  | 13e | Describe any methods used to explore possible causes of heterogeneity among study results (e.g. subgroup analysis, meta-regression). | P8: Materials and Methods (Statistical analysis) - Subgroup analysis, meta-regression |
|  | 13f | Describe any sensitivity analyses conducted to assess robustness of the synthesized results. | P7: Materials and Methods (Statistical analysis) - 'Leave one out' sensitivity analysis |
| Reporting bias assessment | 14 | Describe any methods used to assess risk of bias due to missing results in a synthesis (arising from reporting biases). | P7: Materials and Methods (Statistical analysis) - Funnel plot, Egger's test, Begg's test |
| Certainty assessment | 15 | Describe any methods used to assess certainty (or confidence) in the body of evidence for an outcome. | (Not applicable - No formal GRADE assessment was mentioned in the main text for this outcome) |
| **RESULTS** | | |  |
| Study selection | 16a | Describe the results of the search and selection process, from the number of records identified in the search to the number of studies included in the review, ideally using a flow diagram. | P8: Results (Study Selection and Characteristic); Fig 1 |
|  | 16b | Cite studies that might appear to meet the inclusion criteria, but which were excluded, and explain why they were excluded. | P8-P9: Results (Study Selection and Characteristic) |
| Study characteristics | 17 | Cite each included study and present its characteristics. | P8-P9: Results (Study Selection and Characteristic); Table 1 |
| Risk of bias in studies | 18 | Present assessments of risk of bias for each included study. | P8-P9: Results (Study Selection and Characteristic); S8-S9 Table (NOS Scores) |
| Results of individual studies | 19 | For all outcomes, present, for each study: (a) summary statistics for each group (where appropriate) and (b) an effect estimate and its precision (e.g. confidence/credible interval), ideally using structured tables or plots. | Fig 2 (Tooth brushing), Fig 4 (Tooth loss) |
| Results of syntheses | 20a | For each synthesis, briefly summarise the characteristics and risk of bias among contributing studies. | P9: Results (Association between...); Fig 2 and 4, Table 1 |
|  | 20b | Present results of all statistical syntheses conducted. If meta-analysis was done, present for each the summary estimate and its precision (e.g. confidence/credible interval) and measures of statistical heterogeneity. If comparing groups, describe the direction of the effect. | P9-P10: Results; Table 1; Fig 3 and 5 |
|  | 20c | Present results of all investigations of possible causes of heterogeneity among study results. | P9-P10: Results; Supplementary Fig 2 and 4 |
|  | 20d | Present results of all sensitivity analyses conducted to assess the robustness of the synthesized results. | P9-P10: Results; S1 and S3 Fig (Funnel plots, Egger's/Begg's tests) |
| Reporting biases | 21 | Present assessments of risk of bias due to missing results (arising from reporting biases) for each synthesis assessed. | P9-P10: Results; Statement of "no publication bias" |
| Certainty of evidence | 22 | Present assessments of certainty (or confidence) in the body of evidence for each outcome assessed. | (Not applicable - No formal GRADE assessment was performed) |
| **DISCUSSION** | | |  |
| Discussion | 23a | Provide a general interpretation of the results in the context of other evidence. | P10-P13: Discussion |
|  | 23b | Discuss any limitations of the evidence included in the review. | P13: Discussion |
|  | 23c | Discuss any limitations of the review processes used. | P13: Discussion |
|  | 23d | Discuss implications of the results for practice, policy, and future research. | P13: Discussion |
| **OTHER INFORMATION** | | |  |
| Registration and protocol | 24a | Provide registration information for the review, including register name and registration number, or state that the review was not registered. | P5: Materials and Methods (Literature search) - PROSPERO registration number |
|  | 24b | Indicate where the review protocol can be accessed, or state that a protocol was not prepared. | P5: Materials and Methods (Literature search) - PROSPERO registration number |
|  | 24c | Describe and explain any amendments to information provided at registration or in the protocol. | P5: Materials and Methods (Literature search) - PROSPERO registration number |
| Support | 25 | Describe sources of financial or non-financial support for the review, and the role of the funders or sponsors in the review. | P14: Declarations (Financial Disclosure Statement) |
| Competing interests | 26 | Declare any competing interests of review authors. | P14: Declarations (Conflicting interests) |
| Availability of data, code and other materials | 27 | Report which of the following are publicly available and where they can be found: template data collection forms; data extracted from included studies; data used for all analyses; analytic code; any other materials used in the review. | P14: Declarations (Data Availability) |

*From:*  Page MJ, McKenzie JE, Bossuyt PM, Boutron I, Hoffmann TC, Mulrow CD, et al. The PRISMA 2020 statement: an updated guideline for reporting systematic reviews. BMJ 2021;372:n71. doi: 10.1136/bmj.n71. This work is licensed under CC BY 4.0. To view a copy of this license, visit <https://creativecommons.org/licenses/by/4.0/>

**S7 Table General characteristics of included studies**

| **Author (year)** | **Study Size (patiente/size)** | **Location** | **Study design** | **Setting** | **Adjusted smoking** | **Sex#** | **Diagnosis of gastric cancer** |
| --- | --- | --- | --- | --- | --- | --- | --- |
| **Abnet CC, 2005** | **179/29124** | **America / Europe** | **Cohort** | **Population based** | **No** | **M** | **non-cardia adenocarcinoma** |
| **Akio Hiraki, 2008** | **702/1404** | **Asian** | **Case-control** | **Hospital-based** | **Yes** | **M&F** | **Gastric** |
| **Chun-Han Lo, 2021** | **238/148144** | **America / Europe** | **Cohort** | **Health Professionals** | **Yes** | **M&F** | **Gastric** |
| **Daiki Kobayashi, 2019** | **538/71449** | **Asian** | **Cohort** | **Hospital-based** | **No** | **M&F** | **Gastric cancer** |
| **Kang EJ, 2023** | **3223/200170** | **Asian** | **Cohort** | **Population based** | **Yes** | **M&F** | **Gastric** |
| **Lee K, 2020** | **3587/150774** | **Asian** | **Cohort** | **Population based** | **Yes** | **M&F** | **Gastric** |
| **Ndegwa N, 2018** | **152/19831** | **America / Europe** | **Cohort** | **Population based** | **Yes** | **M&F** | **cardia and non-cardia** |
| **Shakeri R, 2013** | **309/613** | **Asian** | **Case-control** | **Population based** | **No** | **M&F** | **Gastric adenocarcinoma** |
| **Watabe K, 1998** | **242/484** | **Asian** | **Case-control** | **Hospital-based** | **No** | **M&F** | **Gastric** |
| **Yano Y, 2021** | **398/49979** | **Asian** | **Cohort** | **Population based** | **No** | **M&F** | **Gastric adenocarcinomas** |
| **Yano Y, 2021** | **2327/29358** | **Asian** | **Cohort** | **Population based** | **Yes** | **M&F** | **cardia and non-cardia** |
| **Zhang T, 2022** | **901/1972** | **Asian** | **Case-control** | **Population based** | **No** | **M&F** | **cardia and non-cardia** |

**NA, not available; M, male; W, female**

**S7 Table. General characteristics of included studies**

| **Author (year)** | **Outcome ascertainmen** | **Assessment of oral health** | **Definition of oral health** |
| --- | --- | --- | --- |
| **Abnet CC, 2005** | **ICD-10 codes** | **Questionnaire** | **Tooth loss included 0-10, 11-31, and all tooth** |
| **Akio Hiraki, 2008** | **ICD-10 codes** | **Self-administered questionnaire** | **Remaining tooth 0, 1–8, 9–20, and ≥21,** |
| **Chun-Han Lo, 2021** | **medical records and pathology reports.** | **Questionnaire** | **The number of missing tooth ≥2，1, and ≤0** |
| **Daiki Kobayashi, 2019** | **ICD-10 codes, and participants’ self-report** | **Self-administered questionnaire** | **the number of tooth brushings: not every day, once a day, once to twice a day, and every after meal** |
| **Kang EJ, 2023** | **ICD-10 codes and V193 code** | **tooth missing ascertained by dentists** | **whether missing tooth** |
| **Lee K, 2020** | **ICD-10 codes** | **Tooth brushing by self-reported questionnaire and tooth missing ascertained by dentists** | **the number of daily tooth brushings: 0–1 time, 2 times and ≥3 per day. The number of missing tooth 0, 1–7, 8–14, and ≥15,** |
| **Ndegwa N, 2018** | **ICD-10 codes** | **professional clinical examination** | **Number of tooth** |
| **Shakeri R, 2013** | **histologically provened** | **the physical exam by dentist-trained health personnel** | **the frequency of tooth brushings: daily, less than daily and never. The number of missing tooth 32, 25–31,19–24, and ≤12** |
| **Watabe K, 1998** | **Pathologically confirmed** | **Questionnaire** | **Brush tooth, and lack of tooth ≥ 10** |
| **Yano Y, 2021** | **ICD-10 codes and pathology reports** | **Questionnaire** | **Frequency of tooth brushing daily, nondaily and never； tooth loss Q4, Q3, Q2, Q1 VS expected or fewer than expected** |
| **Yano Y, 2021** | **International Endpoints Review Committee** | **Questionnaire and clinical examination** | **Quartiles of Excess tooth loss** |
| **Zhang T, 2022** | **Endoscopy and pathological** | **ascertained by dentists** | **Tooth loss after 20 yr, None, 1–5, and ≥6; frequency of toothbrushing, 0–1 time, and ≥2 per day** |

**S8 TABLE. Quality Scores of Case-Control Studies Using Newcastle-Ottawa Scale**

| **Case-Control Studies** | **Selection** | | | |  | **Comparability** |  | **Exposure** | | | **Total** |
| --- | --- | --- | --- | --- | --- | --- | --- | --- | --- | --- | --- |
|  | **Adequate Definition of Cases** | **Representativeness of Cases** | **Selection of Controls** | **Definition of Controls** |  | **Control for Important Factor or Additional Factor** |  | **Ascertainment of Exposure (Blinding)** | **Same Method of Ascertainment for Participants** | **Nonresponse Rate** |  |
| Shakeri R, 2013 | ★ | ★ | ☆ | ★ |  | ★★ |  | ☆ | ★ | ☆ | 6 |
| Zhang T, 2022 | ★ | ★ | ★ | ★ |  | ★★ |  | ★ | ★ | ★ | 9 |
| Watabe K, 1998 | ★ | ☆ | ☆ | ★ |  | ★ |  | ☆ | ★ | ☆ | 4 |
| Akio Hiraki, 2008 | ☆ | ★ | ☆ | ★ |  | ★★ |  | ☆ | ★ | ☆ | 5 |

**S9 TABLE. Quality Scores of Cohort Studies Using Newcastle-Ottawa Scale**

| **Cohort Studies** | **Selection** | | | |  | Comparability | | Outcome | | | **Total** |
| --- | --- | --- | --- | --- | --- | --- | --- | --- | --- | --- | --- |
|  | **Representativeness of the Exposed Cohort** | **Selection of Non Exposed Cohort** | **Ascertainment of Exposure** | **Outcome of Interest Was Not Present at Start of Study** |  | **Control for Important Factor or Additional Factor** |  | **Assessment of Outcome** | **Follow-up Long Enough for Outcomes to Occur** | **Adequacy of Follow-up of Cohorts** |  |
| Abnet CC, 2005 | ★ | ★ | ★ | ★ |  | ★ |  | ★ | ★ | ★ | 8 |
| Ndegwa N, 2018 | ★ | ★ | ★ | ★ |  | ★★ |  | ★ | ★ | ★ | 9 |
| Chun-Han Lo, 2021 | ☆ | ★ | ★ | ★ |  | ★★ |  | ★ | ★ | ☆ | 7 |
| Yano Y, 2021(Iran) | ★ | ★ | ★ | ★ |  | ★★ |  | ★ | ★ | ☆ | 8 |
| Yano Y, 2021(China) | ★ | ★ | ★ | ★ |  | ★★ |  | ★ | ★ | ★ | 9 |
| Daiki Kobayashi, 2019 | ☆ | ★ | ☆ | ★ |  | ★ |  | ★ | ★ | ★ | 7 |
| Kang EJ, 2023 | ★ | ★ | ★ | ★ |  | ★★ |  | ★ | ★ | ★ | 9 |
| Lee K, 2020 | ★ | ★ | ☆ | ★ |  | ★★ |  | ★ | ★ | ★ | 8 |


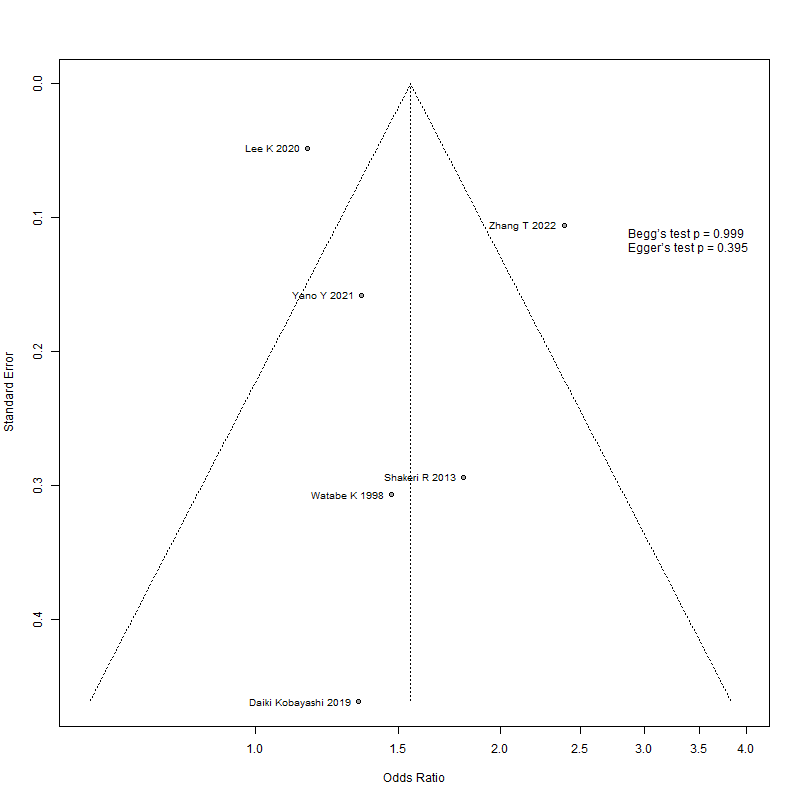
**S1 Fig Funnel plot for the association between tooth brushing and gastric cancer**


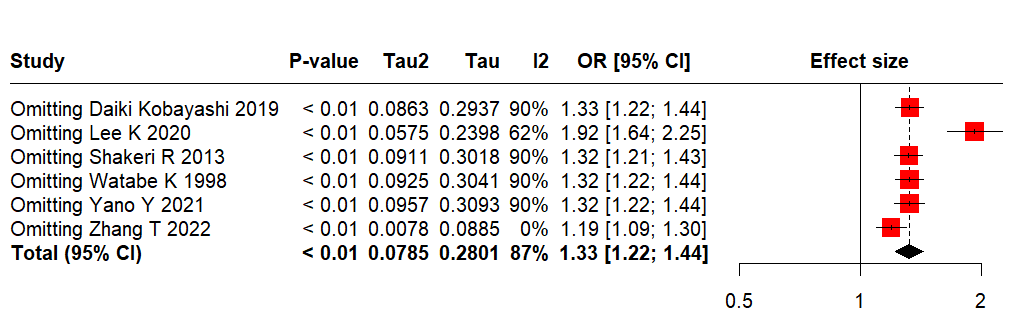
**S2 Fig Sensitivity analysis for the association between tooth brushing and gastric cancer**

**The OR and 95% CI were determined by omitting each study from the pool of eligible studies.**


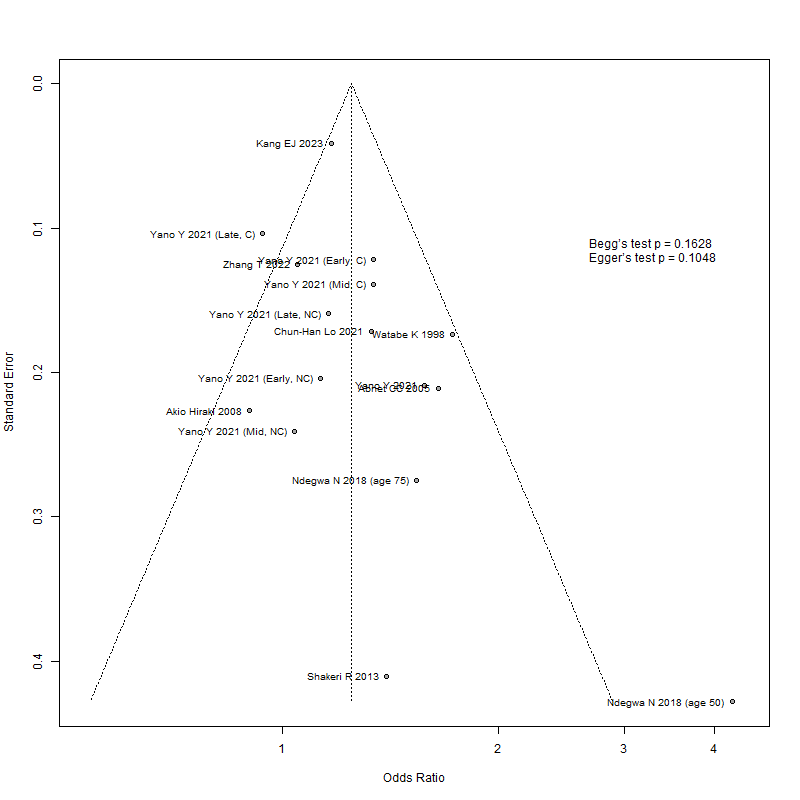


**S3 Fig Funnel plot for the association between tooth missing and gastric cancer**


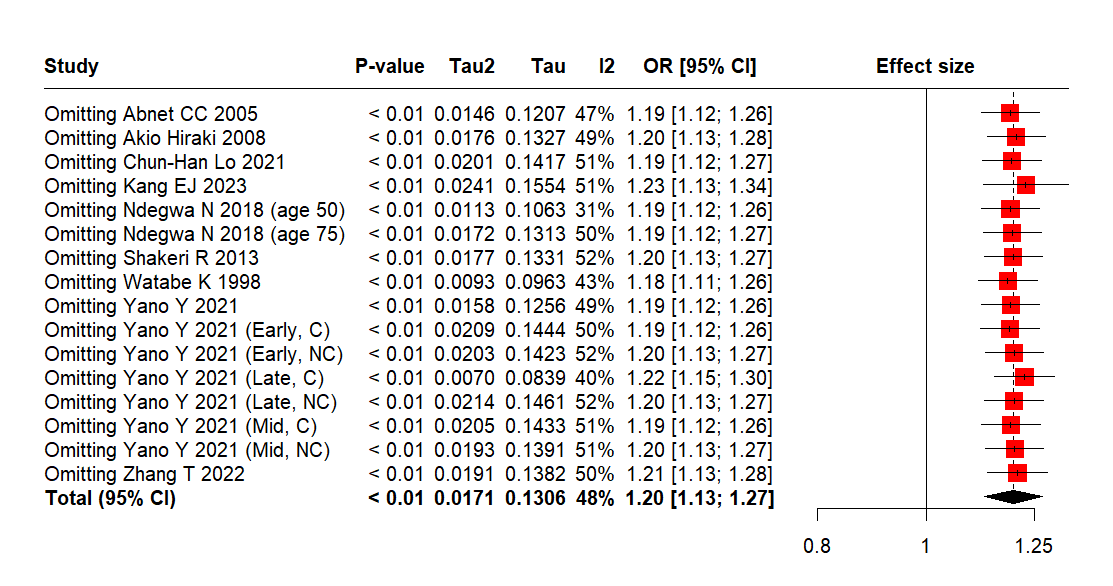


**S4 Fig Sensitivity analysis for the association between tooth brushing and gastric cancer**

**The OR and 95% CI were determined by omitting each study from the pool of eligible studies.**
